# Prospective Study of Proton Therapy for Lung Cancer Patients with Poor Lung Function or Pulmonary Fibrosis

**DOI:** 10.3390/cancers14061445

**Published:** 2022-03-11

**Authors:** Jae Myoung Noh, Hongseok Yoo, Woojin Lee, Hye Yun Park, Sun Hye Shin, Hongryull Pyo

**Affiliations:** 1 Department of Radiation Oncology, Samsung Medical Center, Sungkyunkwan University School of Medicine, Seoul 06351, Korea; rodrno@skku.edu (J.M.N.); wj0616.lee@samsung.com (W.L.); 2 Division of Pulmonary and Critical Care Medicine, Department of Medicine, Samsung Medical Center, Sungkyunkwan University School of Medicine, Seoul 06351, Korea; hongseok.yoo@samsung.com (H.Y.); hyeyunpark@skku.edu (H.Y.P.); fresh.shin@samsung.com (S.H.S.)

**Keywords:** radiotherapy, interstitial lung disease, idiopathic pulmonary fibrosis, lung cancer, proton beam therapy

## Abstract

**Simple Summary:**

Underlying lung disease can affect the pulmonary toxicity after radiotherapy for lung cancer, but treatment outcomes after proton beam therapy (PBT) for lung cancer patients with underlying lung disease have been limited to small retrospective studies. In this prospective study, we aimed to assess pulmonary toxicity following PBT for lung cancer with poor lung function or pulmonary fibrosis. We found that idiopathic pulmonary fibrosis (IPF) was associated with severe pulmonary toxicity and poor survival even after PBT, while PBT seems to be a safe treatment modality for lung cancer patients with chronic obstructive pulmonary disease.

**Abstract:**

PBT has a unique depth–dose curve with a Bragg peak that enables one to reduce the dose to normal lung tissue. We prospectively enrolled 54 patients with non-small cell lung cancer treated with definitive PBT. The inclusion criteria were forced expiratory volume in 1 s (FEV1) ≤ 1.0 L or FEV1 ≤ 50% of predicted or diffusing capacity of the lungs for carbon monoxide (DLco) ≤ 50%, or pulmonary fibrosis. The primary endpoint was grade ≥ 3 pulmonary toxicity, and secondary endpoints were changes in pulmonary function and quality of life. The median age was 71.5 years (range, 57–87). Fifteen (27.8%) and fourteen (25.9%) patients had IPF and combined pulmonary fibrosis and emphysema, respectively. The median predicted forced vital capacity (FVC), FEV1, and DLco were 77% (range, 42–104%), 66% (range, 31–117%), and 46% (range, 23–94%), respectively. During the follow-up (median, 14.7 months), seven (13.0%) patients experienced grade ≥ 3 pulmonary toxicity. Seven months after the completion of PBT, patients with IPF or non-IPF interstitial lung disease (ILD) experienced a decrease in the FVC but the decrease in DLco was not significant. Under careful monitoring by pulmonologists, PBT could be a useful treatment modality for lung cancer patients with poor lung function or pulmonary fibrosis.

## 1. Introduction

Although surgical resection is the treatment of choice for patients with early-stage non-small cell lung cancer (NSCLC), definitive radiation therapy (RT) is recommended as an alternative to surgery for patients unable to undergo resection, including older patients and those with a poor performance status, impaired cardiopulmonary function, or comorbidities [1,2]. For patients with locally advanced disease, multimodality treatment including definitive concurrent chemoradiotherapy (CCRT) is widely applied [1,3]. Pulmonary toxicity, including radiation pneumonitis (RP), is one of the most common treatment-related complications following RT, and underlying lung disease can affect pulmonary toxicity [4,5,6]. Chronic obstructive pulmonary disease (COPD) and idiopathic pulmonary fibrosis (IPF) are well-known pulmonary comorbidities that are associated with an increased risk of lung cancer [7,8,9], but the impact of COPD and IPF on treatment outcomes after RT has not been fully examined [4]. An increased risk of severe radiation-related pulmonary toxicity was demonstrated in patients with IPF [4,6,10].

Pulmonary function could be affected by definitive RT, and poor baseline pulmonary function might adversely affect the survival or pulmonary toxicity [11,12,13]. Therefore, lung cancer patients with poor pulmonary function are also vulnerable as patients with underlying lung disease. Proton beam therapy (PBT) has a unique depth–dose curve with a Bragg peak that enables reduction of the dose to the surrounding normal tissues, such as normal lung tissues [14,15]. Although several studies have reported treatment outcomes after PBT for lung cancer [16,17], treatment outcomes in patients with underlying lung diseases, such as COPD, IPF, and interstitial lung disease (ILD), have been limited to small retrospective studies [18,19]. Because baseline pulmonary function of forced expiratory volume in one second (FEV1) ≥ 1.0 L is one of the eligibility criteria in randomized trials of passive scattering PBT and intensity-modulated radiation therapy (IMRT) [16], the role of PBT in patients with poor pulmonary function could not be assessed. 

Our previous retrospective study showed that PBT was associated with tendencies of better survival and decreased incidences of severe treatment-related toxicity compared to X-ray RT in patients with IPF, but the differences were not statistically significant because of the limited number of patients [18]. Because of the limited capacity and waiting time for PBT, most patients with locally advanced NSCLC received IMRT in our institution [20]. Patients treated with PBT had worse pulmonary function at baseline compared to those treated with IMRT. Considering PBT might play a role in these vulnerable patients in terms of sparing normal lung tissue, we launched a single-arm phase II study of PBT for lung cancer with poor pulmonary function or underlying pulmonary disease to assess pulmonary toxicity and changes in pulmonary function following PBT.

## 2. Materials and Methods

### 2.1. Study Design and Patients

This study was approved by the Institutional Review Board of the Samsung Medical Center (2018-06-002). All participants provided written informed consent. Eligible patients were older than 20 years of age, had pathologically or clinically diagnosed NSCLC that was unsuitable for surgical resection as evaluated by thoracic surgeons, and one of the following: FEV1 ≤ 1.0 L, FEV1 ≤ 50% predicted or diffusing capacity of lungs for carbon monoxide (DLco) ≤ 50%, or pulmonary fibrosis. Patients were required to have an Eastern Cooperative Oncology Group performance status of 0–2. Patients with recurrent or metastatic disease, history of prior RT to the chest, or history of another malignancy within 2 years were excluded. Induction chemotherapy or CCRT was allowed. 

### 2.2. Procedures

Pretreatment assessments consisted of medical history and physical examination, Charlson comorbidity index, blood tests, lung perfusion scans if available, and quality of life (QOL) questionnaires. Pulmonary function tests (PFTs) included spirometry and DLco measurement using a Vmax 22 system (SensorMedics, Yorba Linda, CA, USA) according to the criteria established by the American Thoracic Society/European Respiratory Society [21,22]. The clinical stages according to the 8th American Joint Committee on Cancer staging manual were based on chest computed tomography (CT), 18fluoro-deoxyglucose (FDG) positron emission tomography (PET), and brain magnetic resonance imaging, if needed. Histologic confirmation was strongly recommended. When diagnostic procedures for pathologic confirmation were difficult, risky, or failed because of patients’ underlying lung disease and/or tumor location, lung cancer was diagnosed clinically with serial CT findings and PET-CT under multidisciplinary discussion including a pulmonologist, radiologist, thoracic surgeon, and radiation oncologist.

Careful history taking and serological tests, as well as a review of CT patterns, were implemented for the diagnosis and classification of ILD [23,24]. IPF was diagnosed according to the diagnostic criteria, which requires the exclusion of known causes and the presence of distinct features of CT and/or pathologic patterns [23]. COPD was defined as a pre-bronchodilator FEV1/forced vital capacity (FVC) < 0.70. Combined pulmonary fibrosis and emphysema (CPFE), a recently introduced disease entity, was diagnosed when diffuse fibrosis with emphysema was present on CT along with abnormal gas exchange [25].

For treatment planning, four-dimensional simulation CT scans at a thickness of 2.5 mm were acquired. The gross tumor volume (GTV) was defined as the volume of the tumor identified based on all available clinical information. When tumor motion was less than 1 cm, the internal target volume (ITV) was delineated by combining all GTVs on each respiratory phase, and a 5-mm margin from the ITV was added to generate the clinical target volume (CTV). The margin was modified in accordance with the adjacent organs if necessary, and elective irradiation to the uninvolved nodal region was not applied. The planning target volume was delineated by adding a 5 mm margin to the CTV to account for uncertainties during setup. Breath-hold or respiratory gating was applied for patients with tumor motion ≥ 1 cm. The prescribed dose was 60 GyE or higher, and dose fractionation was individualized according to the tumor size, location, and stage [20,26]. For peripherally located, small-sized tumors, 60 GyE in 4 fractions was prescribed. For tumors close to mediastinal structure or chest wall, 64 GyE in 8 fractions was prescribed. For centrally located tumors (distance from proximal bronchus < 2 cm), 60–69 GyE with 3 or 4 GyE per fraction was prescribed in a PBT-alone case. In the case of definitive CCRT for stage III NSCLC, 61.6–66 GyE in 28–30 fractions was prescribed. The second CT simulation and adaptive re-plan allowed us to accommodate tumor regression and reduce the normal tissue toxicity, especially for patients receiving longer (≥l5) fractions. The planning criteria for organs at risk were as follows, where VD is defined as the percentage of the volume receiving more than D GyE: Both lungs V5 < 65%, V20 < 35%, mean dose to the lung < 20 GyE, mean dose to the esophagus < 34 GyE, mean dose to the heart < 26 GyE, and maximum dose to the spinal cord < 50 GyE. RayStation (RaySearch Laboratories, Stockholm, Sweden) was used for treatment planning. The relative biological effectiveness of PBT was considered a fixed value of 1.1. PBT was delivered using a proton therapy system at our institution (Sumitomo, Niihama, Japan) [27]. The continuous line scanning method or passive scattering with the wobbling method was applied, as described previously [28]. PBT plans were calculated under the Monte Carlo algorithm or pencil beam algorithm [14]. The details of PBT techniques for enrolled patients are summarized in Table S1, and an example of the PBT plan was shown in Figure S1. Daily image guidance was performed with cone-beam CT provided by VeriSuite (MedCom, Darmstadt, Germany) before each treatment session. 

### 2.3. Assessments and Statistical Analysis

Patients were assessed weekly during PBT, including a progress note, physical examination, chest radiography, and RT toxicity. Patients were assessed 1 month and 4 months after the completion of PBT and then every 3 months for 2 years. Development or worsening of respiratory symptoms or radiologic changes were monitored. Chest CT and PFT were obtained at every visit, and FDG-PET scans were obtained 4 months after the completion of PBT. The QOL questionnaire, using the European Organization for Research and Treatment of Cancer Quality of Life-Core 30 (EORTC-QLQ-C30), was completed at 1, 4, and 13 months after the completion of PBT. When pulmonary toxicities of treatment were suspected, patients were thoroughly evaluated by pulmonary physicians. For the exclusion of other possibilities such as infection, exacerbation of underlying pulmonary diseases, or cancer progression, sputum culture and virus polymerase chain reaction tests, chest CT, pulmonary function tests, or a bronchoscopy were performed according to the clinician’s discretion. 

The primary endpoint of this study was grade 3 or higher pulmonary toxicity according to the Common Terminology Criteria for Adverse Events, v4.03. The secondary endpoints included changes in the pulmonary function and QOL. Changes in the percentage of the predicted FVC, FEV1, and DLco from baseline to 7 months after PBT were analyzed with a ranked analysis of covariance (ANCOVA), with the average standardized rank baseline value as a covariate. The Hochberg test was used for post-hoc analysis between groups. QOL subscales were calculated using the EORTC scoring manual [29]. Sample size calculation was based on the assumption that given a two-sided α-value of 0.05 and power of 0.80, the incidences of grade 3 or higher pulmonary toxicity would be 25% and 10% after X-ray treatment and PBT, respectively [4,18]. We assumed an accrual rate of three patients per month and an additional follow-up period of 1 year. Therefore, the required sample size was 50 patients. Considering a 10% dropout rate, the total planned sample size was 55 patients.

Overall survival (OS) was calculated using the Kaplan–Meier method from the start date of PBT to the date of death or the last follow-up and compared between subgroups using the log-rank test. Statistical significance was set at *p* < 0.05. Statistical analysis was performed using SPSS (version 27.0; IBM Corporation, Armonk, NY, USA).

## 3. Results

### 3.1. Baseline Characteristics and PBT

A total of 55 patients were recruited between August 2018 and January 2020. After excluding one patient who did not complete the planned PBT, 54 patients were included in the analysis. The clinical characteristics of the 54 patients are summarized in Table 1. The median age was 71.5 years (range, 57–87 years), and the majority of patients were men (92.6%). The Eastern Cooperative Oncology Group performance status was 0–1 in 53 (98.1%) patients, and the remaining one patient had a performance status of 2. Half of the patients had stage I disease, and one-third of the patients had stage III disease. Among the 18 patients with stage III disease, 13 patients had stage IIIA disease. Squamous cell carcinoma was the most common histology (26 patients, 48.1%), followed by adenocarcinoma (10 patients, 18.5%). The median tumor diameter was 3.3 cm (range, 1.2–19.1 cm). Fifteen (27.8%) patients were clinically diagnosed with lung cancer without histological confirmation. Regarding the underlying lung disease, 19 (35.2%) patients had COPD, 15 (27.8%) patients had IPF, and 16 (29.6%) patients had non-IPF ILD, including CPFE and ILD associated with rheumatoid arthritis or microscopic polyangiitis (MPA). Approximately three-quarters of patients had a DLco ≤ 50%, which represented poor lung function. 

The median and mean CTVs were 133.4 cm^3^ and 183.8 cm^3^, respectively. For patients with early-stage disease, 64 GyE in 8 fractions (*n* = 14, 25.9%) and 60 GyE in 4 fractions (*n* = 13, 24.1%) were the most frequently prescribed dose schedule, and 66 GyE in 30 fractions was the most frequently adopted dose schedule in patients with locally advanced disease (*n* = 10, 18.5%; 9 of them received CCRT) [20]. Overall, the median total dose of 64.0 GyE (range, 60.0–70.4 GyE) with a fraction dose of 7.0 GyE (range 2.2–15.0 GyE) was delivered. The median biologically effective dose at α/β of 10 Gy was 105.6 GyE_10_ (range, 75.2–150 GyE_10_). As shown in Table 2, the V5, V20, and mean doses to the lung were lower than the planning criteria (65%, 35%, and 20 GyE, respectively). The mean doses to the heart and esophagus were 3.1 GyE (range, 0–18.9 GyE) and 5.9 GyE (range, 0–29.5 GyE), respectively.

### 3.2. Pulmonary Toxicity and Pulmonary Function

The median follow-up duration was 19.8 months (range, 1.1–38.0 months). During the follow-up period, seven patients (13.0%) developed grade 3 or higher pulmonary toxicities (Table 3). Among the fifteen patients with IPF, five experienced grade ≥ 3 pulmonary toxicities. Grade 5 pulmonary toxicity was observed in three patients (5.6%), two with IPF and one with ILD associated with MPA (Table 4). All patients with grade ≥ 3 pulmonary toxicities received corticosteroid therapy. Half of the patients with grade 2 pulmonary toxicities were treated with corticosteroids based on the extent and presence of fibrosis in chest CT and respiratory symptoms.

**Figure 1:**
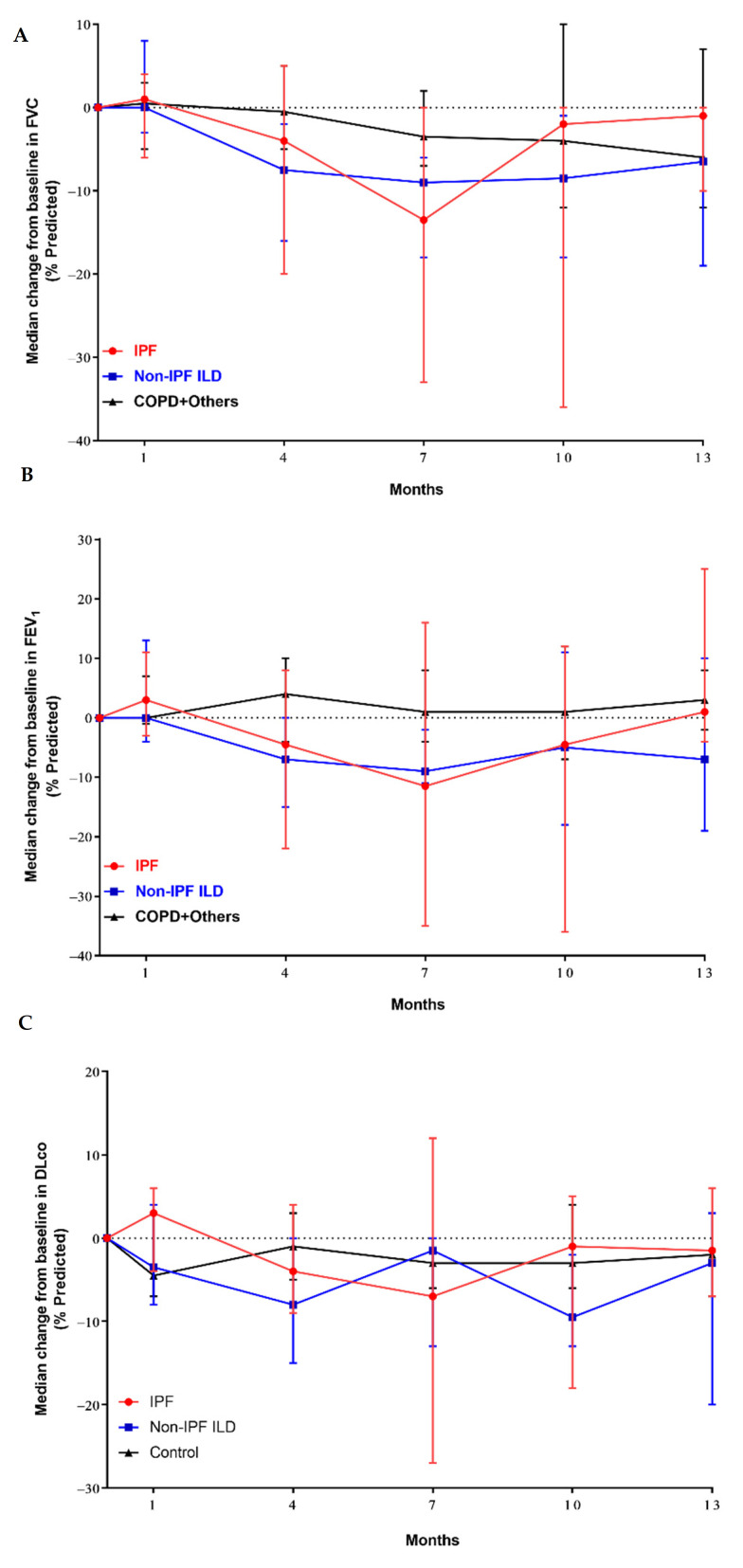
Mean observed changes in (**A**) forced vital capacity, (**B**) forced expiratory volume in 1 s, and (**C**) diffusing capacity of the lungs for carbon monoxide in lung cancer patients with poor lung function. Bars indicate standard errors.

The baseline predicted FVCs in the IPF, non-IPF ILD, and control groups were 75% (range, 42–95%), 81% (range, 54–104%), and 76% (range, 47–103%), respectively. The baseline FVC did not differ among the groups (Kruskal–Wallis, *p* = 0.819). The changes in the predicted FVC from baseline to month 7 were significantly different between the groups in the ranked ANCOVA analysis (*p* = 0.024) (Figure 1A). Post-hoc analysis demonstrated differences in the change in the predicted FVC between the IPF and control groups (Hochberg *p* = 0.089) and between the non-IPF ILD and control groups (Hochberg *p* = 0.048). However, there was no difference in the change in the predicted FVC between the IPF and non-IPF ILD groups (Hochberg *p* > 0.99). The baseline predicted FEV1s in the IPF, non-IPF ILD, and control groups were 80% (range, 44–117%), 73% (range, 56–102%), and 48% (range, 31–78%), respectively. There was a statistically significant difference in the baseline FEV1 among the groups (Kruskal–Wallis, *p* < 0.001). The changes in the predicted FEV1 from baseline to month 7 were not significant in the ranked ANCOVA analysis (*p* = 0.301, Figure 1B). The baseline predicted DLcos in the IPF, non-IPF ILD, and control groups were 45% (range, 28–61%), 47% (range, 23–54%), and 46% (range, 26–94%), respectively (Kruskal–Wallis *p* = 0.518). There was no significant difference in the change in the predicted DLco from baseline to month 7 among the groups in the ranked ANCOVA analysis (*p* = 0.925, Figure 1C).

### 3.3. Quality of Life and Survival

**Figure 2:**
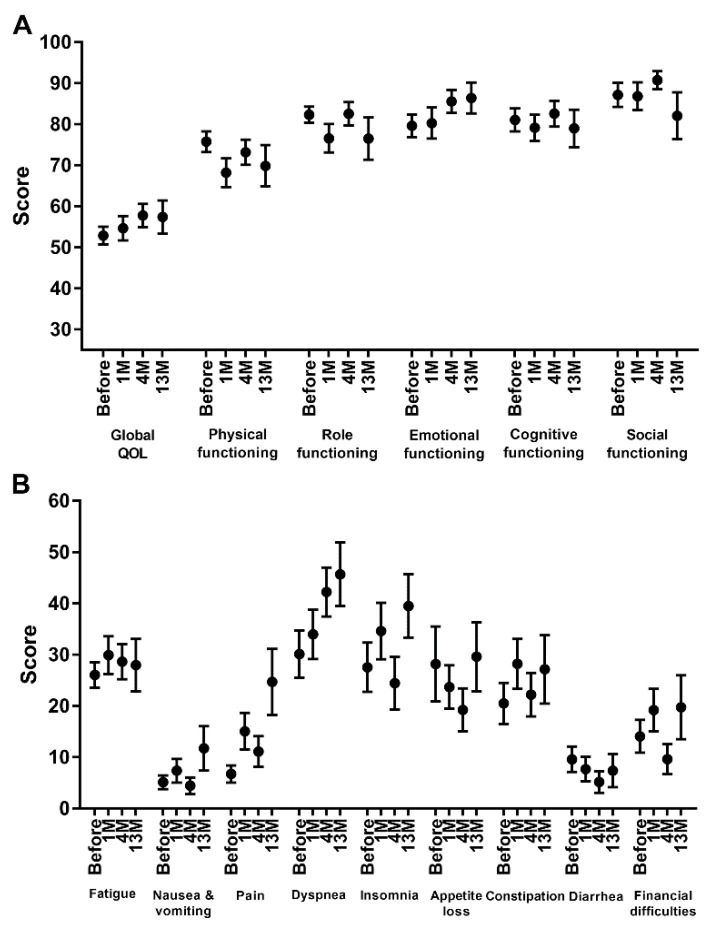
Changes in the mean scores for (**A**) global health status and functioning scales and (**B**) symptom scales before and after proton beat therapy. The bars represent mean scores with standard errors of the mean before proton beam therapy, followed by 1, 4, and 13 months after completion of proton beam therapy, respectively.

Among the 54 patients analyzed, 52 patients completed questionnaires 1 month after PBT; the remaining two patients died before the 1-month follow-up. Forty-five patients completed questionnaires at 4 months, and twenty-five patients completed the questionnaire at 13 months. Figure 2 demonstrates the changes in the mean scores for the EORTC-QLQ-C30 questionnaires. Global health status and functioning scales were well preserved after PBT (Figure 2A). Regarding symptom scales, only dyspnea increased continuously, whereas some symptoms increased at 1 month but had decreased at 4 months (Figure 2B). At 13 months after PBT, the majority of symptom scales, except fatigue, increased compared to that of 4 months.

**Figure 3:**
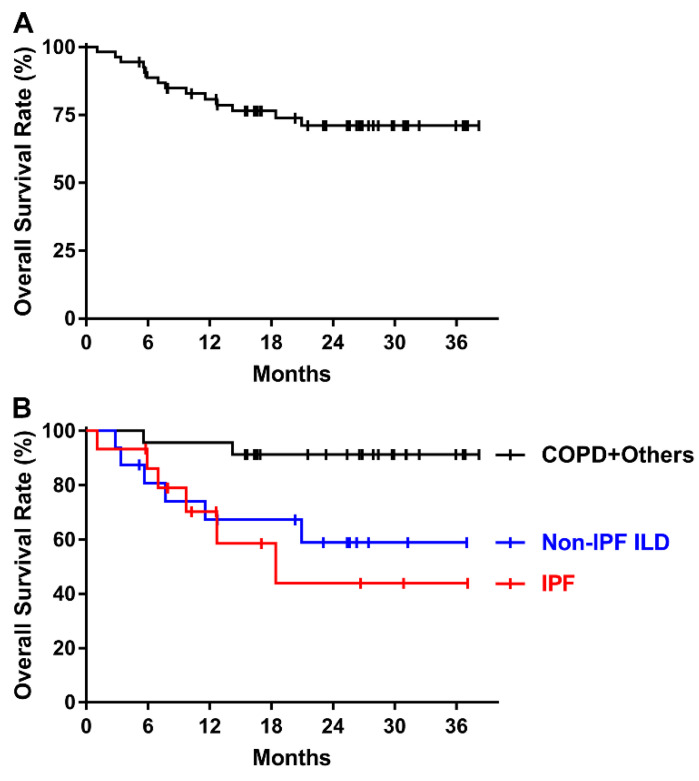
(**A**) Overall survival of all patients. (**B**) Overall survival according to underlying lung disease.

During the follow-up period, 14 patients died after a median of 7.4 months (range, 1.1–21.0 months). The 2-year OS rate was 71.1% (Figure 3A), and it was lower in patients with IPF or non-IPF ILD compared with other patients (43.9%, 58.9%, and 91.3%, *p* = 0.021, Figure 3B). Regarding the patient who died after 1.1 months, the patient was found dead by a neighbor after going to the market. It occurred 24 days after the end of PBT, before the first follow-up visit after PBT. Because there had been no evidence of acute exacerbation of IPF during PBT and we could not know the exact cause of death, we did not consider it as treatment-related death. However, we included him in the survival analysis because he finished the planned PBT.

## 4. Discussion

In this study, we prospectively evaluated the treatment outcomes after PBT for lung cancer patients with poor lung function or pulmonary fibrosis. We had previously assessed the impact of underlying pulmonary disease on treatment outcomes in patients with IPF and early-stage NSCLC who underwent RT [4,18]. There, we saw that the IPF group had a higher incidence of grade ≥ 3 RP (31.8%) than the control, COPD, and CPFE groups (1.6%, 2.3%, and 6.3%, respectively, *p* < 0.001) among patients with stage I-II NSCLC [4]. Although only PBT was applied in the current study, the incidence of grade ≥ 3 RP was 33.3% in patients with IPF (Table 3). Differences in patient populations, such as the inclusion of stage III patients and differences in the control groups, might have influenced the results. However, we must keep in mind that IPF and CPFE were associated with poor survival even after PBT (Figure 3B). The 1-year OS rate in patients with IPF (70.2%) in this study was similar to that in our previous study (66.7%) [18]. There was no treatment-related death after PBT in our previous study, whereas 18.2% of patients treated with X-ray RT died. That study had limitations, including the small sample size and shorter follow-up period in the PBT group. In the current study, the incidence of grade 5 pulmonary toxicity following PBT was 13.3% in patients with IPF (Table 3). Compared with previous studies that reported incidences of grade 5 RP of 26.8–53.8% in patients with interstitial lung changes, the relatively low incidence of grade 5 toxicity in the current study seems to be promising [6,10].

One noteworthy finding of our study is the longitudinal changes in pulmonary function after PBT in patients with various underlying lung diseases. The poor prognosis of IPF is due to progressive fibrosis in the lung parenchyma, a unique characteristic of the disease, which leads to respiratory failure [30]. In addition, a considerable number of patients with COPD also experience a progressive decline in lung function [31]. Given these facts and considering the possibility that RT may result in a decrease in pulmonary function by eliciting radiation-induced lung toxicity such as RP and radiation fibrosis, identifying the impact of RT on serial changes in pulmonary function is essential. However, the results of previous studies on changes in pulmonary function after RT have been contradictory [32,33,34]. Furthermore, risk factors for declines in pulmonary function, including the impact of underlying lung diseases, have not been fully described. Our study showed that the median declines in FVC over 7 months in patients with IPF and non-IPF ILD were greater than those in other patient groups including COPD (IPF vs. non-IPF ILD vs. COPD and others: −68 mL vs. −40 mL vs. −13 mL, Figure 1A), whereas no difference in the FEV1 or DLco was observed among the groups (Figure 1B,C). However, the interpretation of the impact of underlying lung diseases on pulmonary function in our study requires caution. The decline in lung function in patients with IPF and non-IPF ILD may be due to the underlying disease. Furthermore, owing to the low number of patients and high mortality, long-term changes in pulmonary function could not be analyzed. Further studies on the differences in the rates of lung function decline post-RT and in patients with long-term follow-up, as well as assessing the significance of antifibrotic therapy, are warranted. Notably, although baseline FEV1 was significantly lower in the group of COPD and others than IPF or non-IPF ILD, there was no difference in decline among the groups (IPF vs. non-IPF ILD vs. COPD and others: −37 mL vs. −28 mL vs. −1 mL). The results suggest that the impact of RT on lung function of COPD patients may be minimal. Nonetheless, long-term follow-up is necessary to draw firm conclusions.

In our study, 16 (29.6%) patients were diagnosed with non-IPF ILD, including 14 patients with CPFE and two patients with ILD associated with MPA and rheumatoid arthritis. Unlike IPF, whose detrimental effect on the development and prognosis of lung cancer is well documented, the effects of ILDs other than IPF on lung cancer are not fully understood. Although data regarding ILD associated with vasculitis or rheumatoid arthritis are scarce, growing evidence suggests that patients with CPFE are not only at an increased risk of lung cancer development but are also predisposed to treatment-related complications such as pneumonia, acute exacerbation, and persistent air leakage [35,36]. A recent meta-analysis of patients with CPFE and lung cancer reported that CPFE is associated with poorer OS and higher rates of postoperative complications and short-term postoperative mortality than patients with no underlying lung diseases and patients with emphysema alone. These outcomes were similar to those of patients with IPF [35]. The findings of our study are in line with the results of this meta-analysis. Although there was a tendency toward a lower rate of significant RP (grade 3 or more) in patients with non-IPF ILD than in those with IPF (6.3% vs. 33.3%), OS and the magnitude of the decline in pulmonary function were similar between the two groups. The substantial rate of treatment-related toxicities and mortality observed in patients with lung cancer and either CPFE or IPF necessitates the establishment of optimal treatment strategies. Interestingly, in the subgroup analysis of the previously mentioned meta-analysis, the OS between patients with and without CPFE differed only in patients undergoing surgery but not in patients undergoing other treatments. In a study on CPFE and lung cancer by Usui et al., acute lung injury following RT occurred in 16.7% of patients compared to 27.3% and 20.0% following surgery and chemotherapy, respectively. Despite the limitations in the validity of these findings, they suggest that nonsurgical treatment such as RT may be beneficial to patients with ILD and lung cancer. Additional studies are necessary to identify effective and safe treatment modalities for IPF and non-IPF ILD patients with lung cancer. Finally, only one patient from the group of twenty-three patients with COPD and others suffered from RP grade 3 and no patient experienced RP grade 5. Furthermore, the 2-year survival reached up to 91.3%. The results of this study, as well as our previous study, provide compelling evidence that RT may be a safe treatment modality for lung cancer patients with COPD [4].

Although this study has strengths regarding the longitudinal changes in pulmonary function and QOL in patients with poor lung function, there are some potential limitations. First, this was a single-arm study. Therefore, direct comparison with X-ray treatment is not possible. However, randomized studies might be practically and ethically difficult because of the significant risk of severe pulmonary toxicity in patients with ILD. Second, underlying lung disease was heterogeneous, such as IPF, CPFE, and COPD. Additionally, we also included patients with poor pulmonary function. Considering these limitations in terms of patients’ underlying lung disease, we have launched a prospective study of PBT for patients with pulmonary fibrosis (IPF and CPFE) who had poorer OS in the current study. As IPF was associated with an increased incidence of grade ≥3 pulmonary toxicity, including grade 5 toxicity (Table 3 and Table 4), additional efforts need to be made to ameliorate pulmonary toxicity in these patients, such as antifibrotic therapy. Fourth, COPD was defined based on pre-bronchodilator pulmonary function tests. Thus, some of the patients may have been misclassified. However, as post-bronchodilator pulmonary function tests are not routinely performed in patients who receive RT, our data may be more informative for real-world clinical settings. Finally, the majority of our patients with pulmonary toxicities received corticosteroid treatment. Although corticosteroid treatment is generally accepted as a mainstay of therapy, the optimal duration and regimen of treatment are still unknown. Further studies are required to confirm proper management strategies for patients with pulmonary toxicities.

## 5. Conclusions

In conclusion, PBT could be a useful treatment modality for lung cancer patients with poor lung function or pulmonary fibrosis. Careful monitoring by pulmonologists and proper management of pulmonary toxicities and underlying lung diseases following PBT are important, especially in patients with IPF or CPFE.

## Figures and Tables

**Table 1:** Clinical characteristics.

Characteristics	*n* (%)
Age (years)	
Median (range)	71.5 (57–87)
Gender	
Female	4 (7.4%)
Male	50 (92.6%)
Smoking	
Never smoker	3 (5.6%)
Current smoker	12 (22.2%)
Ex-smoker	39 (72.2%)
Charlson Comorbidity index	
0	9 (16.7%)
1	38 (70.4%)
2–3	7 (13.0%)
Clinical stage	
I	27 (50.0%)
II	9 (16.7%)
III	18 (33.3%)
Histology	
Adenocarcinoma	10 (18.5%)
Squamous cell carcinoma	26 (48.1%)
Others	3 (5.6%)
Not proven	15 (27.8%)
Underlying lung disease	
COPD	19 (35.2%)
IPF	15 (27.8%)
CPFE	14 (25.9%)
Other ILD	2 (3.7%)
Others	4 (7.4%)
FEV1	
>50%	38 (70.4%)
≤50%	16 (29.6%)
Median (range)	1.91L (0.80–3.18)
DL_CO_	
>50%	14 (25.9%)
≤50%	40 (74.1%)
Median (range)	46% (23–94)

COPD, chronic obstructive pulmonary disease; IPF, idiopathic pulmonary fibrosis, CPFE, combine pulmonary fibrosis and emphysema; ILD, interstitial lung disease; FEV1, forced expiratory volume in 1 s; DL_CO_, diffusing capacity of the lung for carbon monoxide.

**Table 2:** Dose-volume parameters.

Parameters	Planning Criteria	Planning Results
CTV (cm^3^)		183.8 (9.6–792.9)
CTV_95%_ (%)		95.77 (53.2–100)
CTV_100%_ (%)		88.52 (40.3–99.4)
Total lung		
Mean dose (GyE)	20	7.4 (1.5–16.2)
V5 (%)	65%	19.6 (5.4–43.3)
V20 (%)	35%	13.3 (2.6–31.8)
Heart		
Mean dose (GyE)	26	3.1 (0–18.9)
V40 (%)		3.8 (0–40)
Esophagus		
Maximum dose (GyE)		28.5 (0–76.4)
Mean dose (GyE)	34	5.9 (0–29.5)
Spinal cord		
Maximum dose (GyE)	50	12.9 (0–40.3)

CTV, clinical target volume; V_xx%_ = volume receiving XX% of the prescription dose; V_xxGyE_ = volume receiving more than XX GyE. Values are presented as mean (range).

**Table 3:** Pulmonary toxicity according to underlying lung disease.

Underlying Lung Disease	Grade 0	Grade 1	Grade 2	Grade 3	Grade 5
IPF	2 (13.3%)	5 (33.3%)	3 (20.0%)	3 (20.0%)	2 (13.3%)
CPFE and non-IPF ILD	5 (31.3%)	5 (31.3%)	5 (31.3%)	0	1 (6.3%)
COPD and others	4 (17.4%)	8 (34.8%)	10 (43.5%)	1 (4.3%)	0

IPF, idiopathic pulmonary fibrosis, CPFE, combined pulmonary fibrosis and emphysema; ILD, interstitial lung disease; COPD, chronic obstructive pulmonary disease.

**Table 4:** Clinical characteristics and course of patients with grade 5 pulmonary toxicity.

No	Age	Sex	FEV1	DLCO	Underlying	Stage	MLD	Dose	Survival (Months)
1	65	F	1.59L (73%)	43%	MPA ILD	T3N0	8.6 Gy	60 GyE/15fx	3.4
2	62	F	1.98L (83%)	31%	IPF	T2aN0	10.4 Gy	60 GyE/15fx	6.0
3	69	M	2.07L (65%)	45%	IPF	T2bN0	8.1 Gy	64 GyE/16fx	7.0

FEV1, forced expiratory volume in 1 s; DL_CO_, diffusing capacity of the lung for carbon monoxide; MLD, mean lung dose; MPA, microscopic polyangiitis; ILD, interstitial lung disease; IPF, idiopathic pulmonary fibrosis.

## Data Availability

The datasets generated and analyzed during the current study are not publicly available due to institutional data protection law and confidentiality of patient data but are available from the corresponding author upon reasonable request in person.

## Supplementary Materials

The following supporting information can be downloaded at: https://www.mdpi.com/article/10.3390/cancers14061445/s1, Figure S1: Example of proton beam therapy plan for a patient with T3N0M0 squamous cell carcinoma in the left lower lobe and idiopathic pulmonary fibrosis treated with proton beam therapy to 64 GyE in 8 fractions, Table S1: Characteristics of proton beam therapy.
