# Supplementary material for: Prospective Study of Proton Therapy for Lung Cancer Patients with Poor Lung Function or Pulmonary Fibrosis
Source: Cancers (Basel). 2022 Mar 11;14(6):1445. doi: 10.3390/cancers14061445 (PMC8946495; doi:10.3390/cancers14061445)
Supplement: Supplementary file 1 [file cancers-14-01445-s001.zip › cancers-1612179-supplementary.pdf]

cancers

MDPI

Article

Prospective study of proton therapy for lung cancer patients with poor lung function or pulmonary fibrosis

Jae Myoung Noh, Hongseok Yoo, Woojin Lee, Hye Yun Park, Sun Hye Shin, and Hongryull Pyo

Table S1. Characteristics of proton beam therapy.

Technique	Calculation algorithm	Optimization		Total
		SFO	MFO	
**Continuous line scanning**	**Monte Carlo algorithm**	31	5	36
	**Pencil beam algorithm**	5	0	5
Passive scattering	Pencil beam algorithm	13	0	13
Total		49	5	54

SFO, single-field optimization; MFO, multi-field optimization.

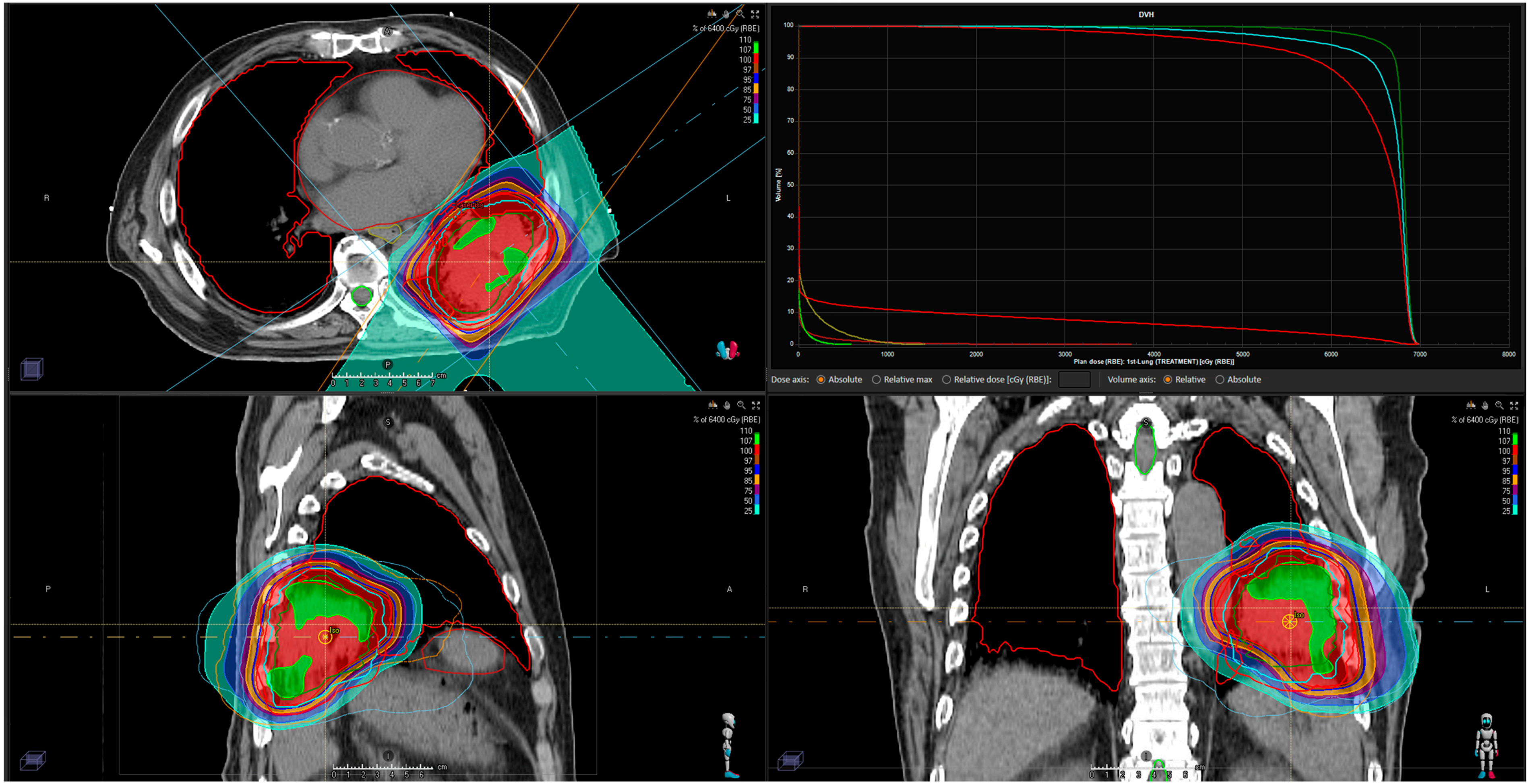

Figure S1. Example of proton beam therapy plan for a patient with T3N0M0 squamous cell carcinoma in the left lower lobe and idiopathic pulmonary fibrosis treated with proton beam therapy to 64 GyE in 8 fractions. Contours including the internal target volume (dark green), clinical target volume (blue), planning target volume (red), spinal cord (green), esophagus (dark yellow), heart (red purple), and both lung (red). Because the tumor was located close to the stomach, target coverage was adjusted to lower dose to the stomach.
